# Case report: Death caused by 1, 3-dichloropropene, a novel fumigant used in China

**DOI:** 10.3389/fpubh.2023.1088296

**Published:** 2023-02-16

**Authors:** Zhiqiang Zhou, Lanlan Guo, Zixin Wen, Ping Dai, Tongyue Zhang, Wenjun Wang, Xiangdong Jian

**Affiliations:** ^1^Department of Occupational and Environmental Health, School of Public Health, Cheeloo College of Medicine, Shandong University, Jinan, Shandong, China; ^2^Department of Poisoning and Occupational Diseases, Emergency Medicine, Cheeloo College of Medicine, Qilu Hospital, Shandong University, Jinan, Shandong, China; ^3^School of Nursing and Rehabilitation, Cheeloo College of Medicine of Shandong University, Nursing Theory and Practice Innovation Research Center of Shandong University, Jinan, Shandong, China

**Keywords:** 1, 3-dichloropropene, fumigant, acute renal failure, brain edema, self-protection

## Abstract

As an efficient and broad-spectrum soil fumigant, 1, 3-dichloropropene is widely used in the control of nematodes, soil pests, and plant pathogens. However, as a volatile chlorine-containing organic compound, 1, 3-dichloropropene is harmful to human health, although no deaths caused by inhalation of 1, 3-dichloropropene have been reported. This article describes the case of a 50-year-old man who died of acute renal failure and brain edema after inhaling 1, 3-dichloropropene at work. This case demonstrates that 1, 3-dichloropropene can be absorbed through the respiratory tract and that exposure to 1, 3-dichloropropene in a confined environment without any protective measures can cause death in humans.

## 1. Introduction

**Figure 1:**
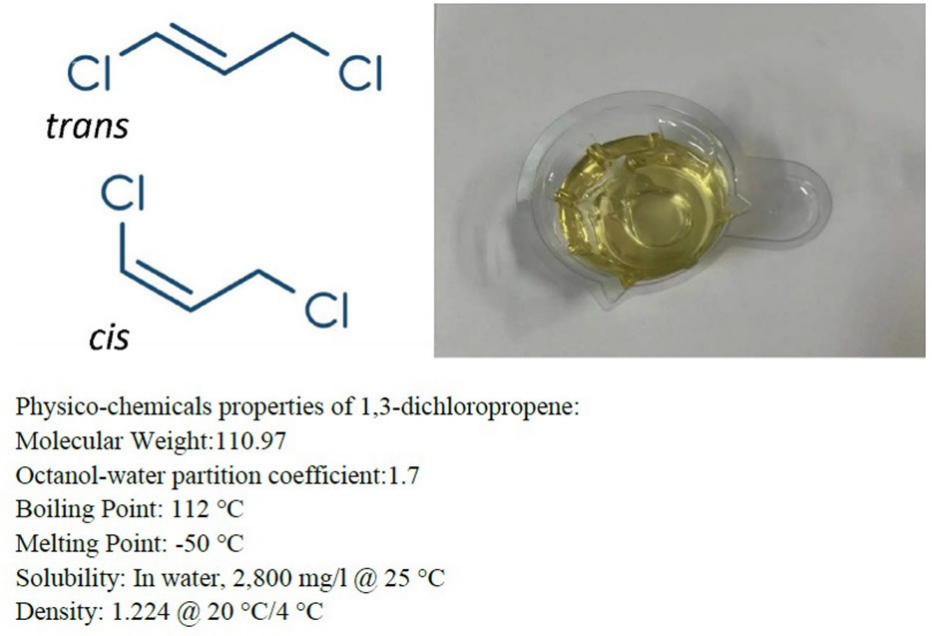
1, 3-dichloropropene.

1,3-dichloropropene (C3H4Cl2; CAS no: 542-75-6) (Figure 1) exists as *cis-* and *trans-* isomers (1). At room temperature, it is colorless or amber, with the excitant odor of chloroform (2, 3). It is a volatile liquid, widely used as a soil fumigant to control nematode pests in agriculture before planting (4). Human skin exposure can cause skin irritation. Meanwhile, inhalation may result in severe signs and symptoms of intoxication, which can cause central nervous system depression (5). Here, we report the case of a patient poisoned by absorption of 1, 3-dichloropropene through the respiratory tract, who presented with acute renal failure, hyperkalemia, and cerebral edema, and who eventually died.

## 2. Case description

**Figure 2:**
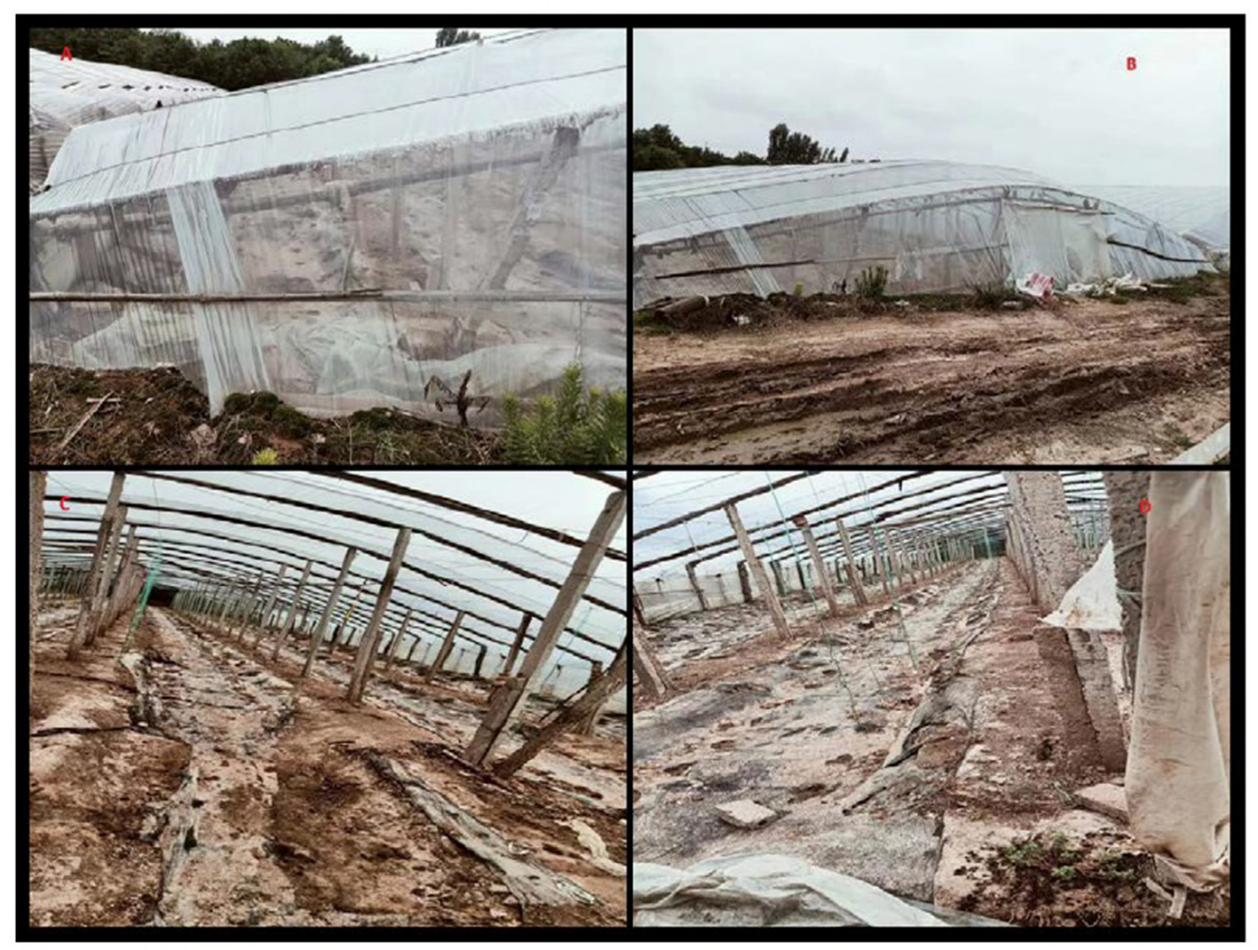
Photo of greenhouse where the patient was working at that time. Picture of **(A, B)** are external photos of greenhouse, picture **(C, D)** are internal photos of greenhouse.

**Figure 3:**
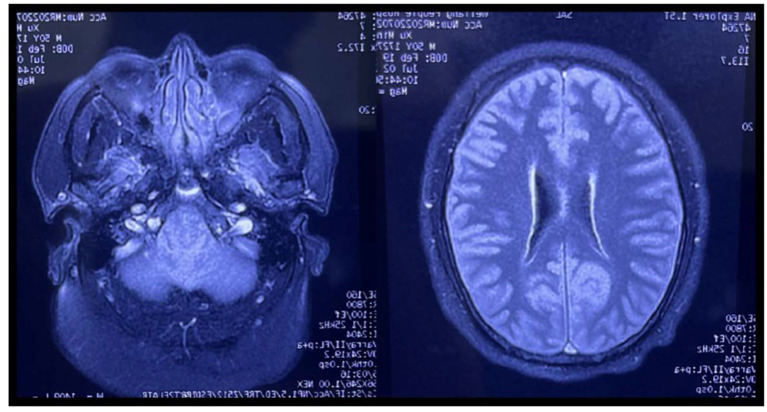
Craniocerebral magnetic resonance examination results of the patient.

A 50-year-old man presented to the emergency department of a local hospital with dizziness and confusion. According to his family, he worked in the family's greenhouse (see Figure 2). The length of the greenhouse is 30 m, the width is 8 m, and the height is 2 m. He entered the planting greenhouse at 10 p.m. without proper ventilation, without wearing respiratory protection, and bare-chested. After wearing a surgical mask at the door of the greenhouse, his wife diluted the 1, 3-dichloropropene (see Figure 1) with water. The dilution process was 500 ml of 1, 3-dichloropropene was added to 25 L of water for stirring dilution, and the dilution ratio was 1:50. Then, his wife irrigated the diluted 1, 3-dichloropropene on the surface of the ground at the door of the greenhouse along with the trench in the field. During this period, the patient was alone in the greenhouse for inspection and smoked an unknown amount of tobacco in the greenhouse until 3 a.m. the next day. After returning home, the patient began to have headaches, dizziness, and other discomforts, and no special treatment was given. On the afternoon of the third day, the patient had blurred vision and unclear speech, as well as other symptoms, and the dizziness was worse than before. At 6 p.m., he was sent to a county People's Hospital by his family because of dizziness, lightheartedness, and unclear expression for 1 day, during which he developed symptoms of irritability, and a head CT examination showed no abnormality. On the fourth day, the patient was transferred to a municipal People's hospital. A head CT examination was performed, and no abnormality was found. Later, the patient was transferred to the ICU ward. On the 5th day, a craniocerebral CT examination was performed, showing unclear sulci and cisternae, slightly higher density, and an unclear third ventricle. On the 6th day, craniocerebral magnetic resonance examination (Figure 3) showed diffuse enlargement of the brainstem, uneven signal, and compression of the fourth ventricle narrowed. The brain tissue exhibited diffuse swelling, the cerebellar tonsil moved down, and the sulci and cisternae were not clear. Bilateral anterior, middle, and posterior cerebral arteries were not clearly visible. The patient was directed to come to our department at 6 p.m.

Upon admission, the patient's status was as follows: endotracheal intubation ventilator-assisted ventilation, ventilator parameters: V-SIMV mode; oxygen concentration, 60%; tidal volume, 450 ml; respiratory rate, 14 times/min; PEEP, 4 cm H_2_O; vital signs were as follows: temperature, 36.6°C; heart rate, 109 bpm; blood pressure, 102/72 mmHg; and oxygen saturation, 94%. Laboratory test results are shown in Table 1. Liver function, coagulation series, myocardial enzymes, and routine blood work showed no abnormalities. After admission, comprehensive treatment was given immediately, including electrocardiogram monitoring, blood oxygen saturation monitoring, ventilator-assisted ventilation, dexamethasone (40 mg, intravenous drip, over 24 h) to reduce the symptoms of cerebral edema, torasemide (20 mg, intravenous injection, twice a day) to promote drainage diuresis, vitamin B1 (100 mg, intramuscular injection, once a day) to maintain the body glucose metabolism, rat nerve growth factor (30 μg, intramuscular injection, once a day) nutrition, and other symptomatic support treatment. On the 2nd day of admission, the 24-h urine volume of the patient was 200 ml, and the heart rate was 125 bpm. The patient was immediately given continuous renal replacement therapy (CRRT) for 8 h, mode was continuous venovenous hemofiltration (CVVH), the blood flow was 160 ml/min, the pre-blood pump (PBP) was 130 ml/h, and the dehydration volume was 300 ml/h. On the third day after admission, the patient's condition worsened, and ventricular fibrillation commenced. Blood pressure was measured at 44/28 mmHg, and then cardiac arrest occurred. Laboratory parameters were as follows: aspartate aminotransferase (AST), 106I U/L; alanine aminotransferase (ALT), 65I U/L; urea nitrogen (BUN), 31.4 mmol/L; creatinine (Cr), 522 μmol/L; creatine kinase (CK), 6,633I U/L; and blood potassium, 8.48 mmol/L. The patient eventually died of acute renal failure, hyperkalemia, and cerebral edema.

**Table 1:** Results of various laboratory tests before and after admission.

**Detection time**	**Day 1**	**Day 3**	**Local hospital**	**Reference value**
White blood cell (10^9^/L)	8.54	11.30	7.44	3.5–9.5
Neutrophils (%)	83.20	83.20	82.40	40–75
Red blood cells (10^12^/L)	4.75	4.90	5.52	4.3–5.8
Hemoglobin (g/L)	148.0	154.0	165	130–175
Mean corpuscular volume (fL)	95.7	92.7	92.9	82–100
Red blood cell specific volume (%)	45.50	45.50	51.3	40.0–50.0
Platelet (10^9^/L)	147	102	190	125–350
Alanine transaminase (IU/L)	34	65	24	9–50
Aspartate aminotransferase (IU/L)	42	106	15	15–40
Total bilirubin (mmol/L)	8	8	6.9	5.0–21.0
Urea nitrogen (mmol/L)	13.6	31.4	6.3	2.30–7.80
Serum creatinine (μmol/L)	165	522	74	62–115
Blood glucose (mmol/L)	18.2	21.3	11.9	3.90–6.10
Creatine kinase (IU/L)	43	6,633	-	55–170
Potassium ion (mmol/L)	3.14	8.48	3.23	3.5–5.5
Sodium ion (mmol/L)	157.0	147	150	135.0–145.0

## 3. Discussion

1, 3-dichloropropene is widely used as a soil fumigant for the control of root-knot nematodes and other soil pests and diseases. In previous studies on the inhalation of 1, 3-dichloropropene in rats, it was observed that 1, 3-dichloropropene is easily absorbed, binds to glutathione (GSH) through glutathione S-transferase (GST), and is excreted rapidly in the urine. The half-life of 1, 3-dichloropropene in the body is short; therefore, it does not accumulate in the body (6). However, when rats were exposed to 1,716 ppm 1, 3-dichloropropene, significant reductions in glutathione levels were observed in the heart, lungs, liver, and testis (7). Although rats can metabolize 1, 3-dichloropropene rapidly upon inhalation at low concentrations, transient exposure to 1, 3-dichloropropene at concentrations above 2,700 ppm can irritate the eyes and nose and cause severe lung, nose, liver, and kidney damage (8). In previous studies, researchers summarized the following symptoms observed in rats after a given concentration of 1, 3-dichloropropene was inhaled over a short period of time: closing eyes, pore expanding, salivation, tears, lethargy, diarrhea, reduced frequency of breathing, and irregular respiratory movement (pulmonary congestion) were observed in animal death and hunched posture, red ears, and tail. Pathological signs of heart failure, acute tubular necrosis of the kidney, and local impact on the respiratory tract were observed (9). Thus, it cannot be ruled out that 1, 3-dichloropropene because of its volatile and highly lipophilic nature absorbs quickly into the body through the respiratory tract and skin, and through the blood–brain barrier in the brain tissue due to high lipid content thereby inhibiting the central nervous system, leading to respiratory and circulatory failure.

In the present case, after understanding the patient's working history before the onset of the disease and the history of the present disease, it was determined that the substance causing the poisoning was 1, 3-dichloropropene. Walking in the damp climate, high temperature, and poorly ventilated greenhouses, when exposed to 1, 3-dichloropropene for a short time, the patient inhaled 1, 3-dichloropropene and quickly experienced dizziness, fatigue, nausea, unconsciousness, breathing difficulties, and other symptoms. Combined with the results of the serological test and brain magnetic resonance imaging, the symptomatic presentation was consistent with the manifestation of acute 1, 3-dichloropropene poisoning.

In conclusion, short-term exposure to a certain concentration of 1, 3-dichloropropene is harmful to the human body in a closed environment. Absorption through the respiratory tract may be followed by passage across the blood-brain barrier, deposition in the brain tissue, then inhibit the central nervous system and cause diffuse brain tissue edema, leading to acute damage to heart, lung, and kidney function, and eventually leading to death.

This case suggests that for patients with dizziness, headache, limb weakness, cognitive dysfunction, and disturbance of consciousness in the early stage, medical staff should record the patient's work history and the chemicals used over the years in detail, combined with typical neuroimaging manifestations, so as to make an early diagnosis. As a volatile organic solvent, 1, 3-dichloropropene can cause respiratory stimulation response and central nervous system inhibition after inhalation, which can cause dyspnea and brain edema. Oxygen inhalation should be given early, and in severe cases, sufficient and long-term dehydrating agent and glucocorticoid treatment should be given early to reduce brain edema and improve the prognosis of patients. In order to reduce the risk of such poisoning events, the pesticide applicators should have read the instructions for the product, understood how to handle the chemical product, ensured that the treated area have sufficient ventilation, and donned appropriate personal protective equipment (skin, eye, and respiratory protection) before entering the working environment. The pesticide applicators should have regular health examinations. The combination of solvent exposure and smoking is likely not conducive to recovery and may exacerbate harm acutely.

## Data availability statement

The original contributions presented in the study are included in the article/supplementary material, further inquiries can be directed to the corresponding authors.

## Ethics statement

Written informed consent was obtained from the individual(s) for the publication of any potentially identifiable images or data included in this article.

## Author contributions

ZZ and XJ investigated the description of the incident. ZZ conceived the study and drafted the manuscript. ZZ, LG, ZW, PD, and TZ supervised data collection. ZZ, WW, and XJ take responsibility for the paper as a whole. All authors contributed substantially to its revision. All authors contributed to the article and approved the submitted version.
